# Supplementary material for: New Epigenetic Modifier Inhibitors Enhance Microspore Embryogenesis in Bread Wheat
Source: Plants (Basel). 2024 Mar 8;13(6):772. doi: 10.3390/plants13060772 (PMC10975478; doi:10.3390/plants13060772)
Supplement: Supplementary file 1 [file plants-13-00772-s001.zip › plants-2814000-supplementary.pdf]

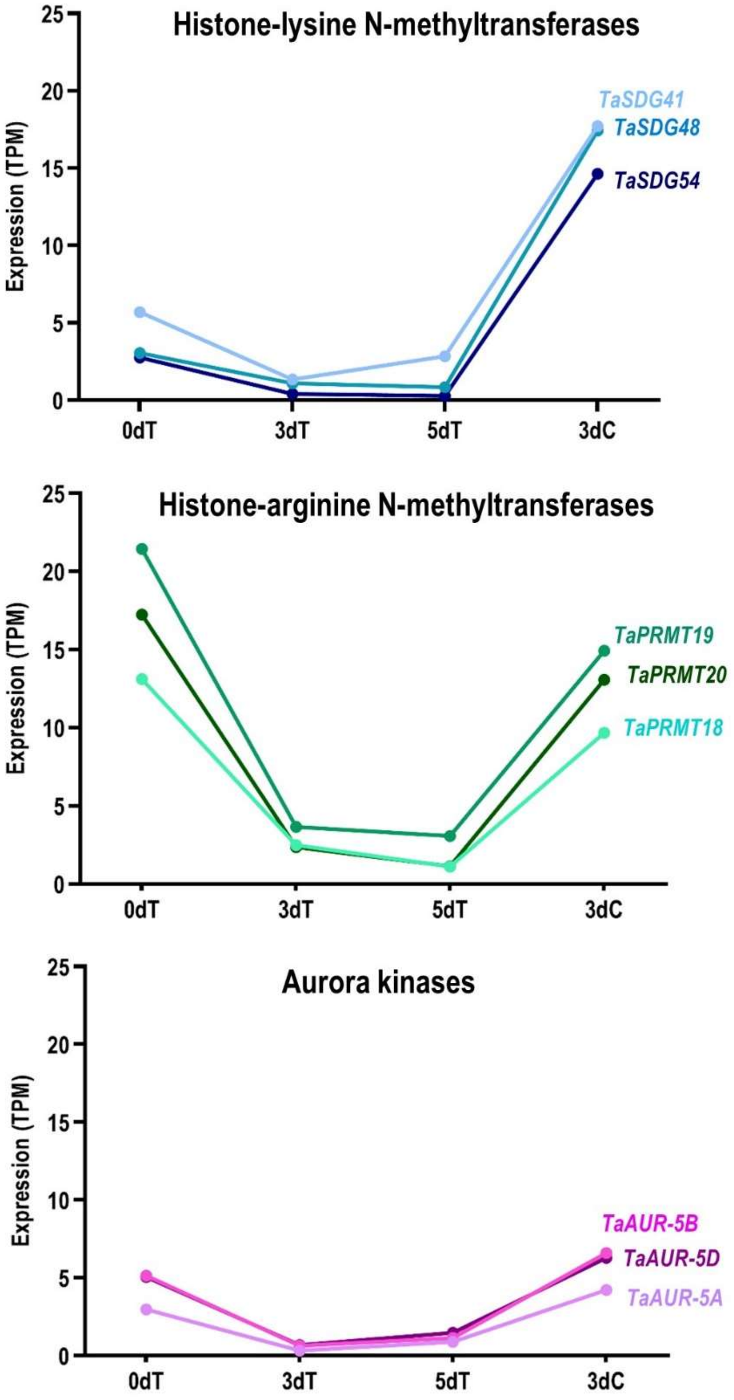


**Supplementary Figure S1**: Gene expression of wheat histone-lysine N-methyltransferases, histone-arginine N-methyltransferases and Aurora kinases before ME induction (0dT), after 3 or 5 days of stress treatment (3dT and 5dT) and after 3 days in culture (3dC) after ME induction. Data based in a wheat RNA-seq analysis (Valero-Rubira et al., unpublished data).

**Supplementary Table S1**: Percentages of microspores, bicellular structures, tricellular structures (both pollen-like structures or embryogenic structures), tetracellular and multicellular embryogenic structures after application of 0.4 μM TSA (L-0.4TSA), Chaetocin (L-0.4Chaetocin), CARM1 inhibitor (L-0.4CARM1I), Aurora Kinase Inhibitor II (L-0.4AUKI-II) and Hesperadin (L-0.4Hesperadin), during a 24-hour stress treatment in SM liquid medium. **A**) after 2 days of culture (2dC); **B**) after 4 days of culture (4dC); **C**) after 10 days of culture (10dC) in Pavon wheat cultivar. L-CM = control in SM liquid medium; L-CM+DMSO= control DMSO in SM medium with 1 % DMSO. Values followed by the same letter within each treatment are not significantly different ($p<0.05$) according to a Chi square test.

| | | | | Embryogenic structures | |
|---|---|---|---|---|---|
| **A) Pavon 2dC (%)** | **Microspores** | **Bicellular** | **Tricellular** | **Tetracellular** | **Multicellular** |
| **L-CM** | 41.0 a | 57.0 ab | 2.0 a | 0.0 a | 0.0 a |
| **L-CM+DMSO** | 43.0 a | 53.6 bc | 3.1 a | 0.3a | 0.0 a |
| **L-0.4TSA** | 26.8 b | 68.9 a | 4.1 a | 0.24 a | 0.0 a |
| **L-0.4Chaetocin** | 34.8 ab | 62.1 ab | 2.9 a | 0.2 a | 0.0 a |
| **L-0.4CARM1I** | 34.7 ab | 6077 ab | 4.0 a | 0.6 a | 0.0 a |
| **L-0.4AUKI- II** | 36.1 ab | 59.3 ab | 3.6 a | 0.9 a | 0.0 a |
| **L-0.4Hesperadin** | 35.8 ab | 60.1 ab | 3.4 a | 0.8 a | 0.0 a |

| | | | Tricellular structures | | Embryogenic structures | |
|---|---|---|---|---|---|---|
| **B) Pavon 4dC (%)** | **Microspores** | **Bicellular** | **Pollen-like** | **Embryogenic** | **Tetracellular** | **Multicellular** |
| **L-CM** | 48.7 a | 48.5 a | 2.7 a | 0.0 b | 0.2 a | 0.0 a |
| **L-CM+DMSO** | 46.3 ab | 48.4 a | 2.1 a | 3.2 ab | 0.0 a | 0.0 a |
| **L-0.4TSA** | 35.1 ab | 56.8 a | 3.4 a | 3.4 ab | 1.0 a | 0.3 a |
| **L-0.4Chaetocin** | 36.8 ab | 58.4 a | 2.8 a | 0.9 ab | 1.1 a | 0.0 a |
| **L-0.4CARM1I** | 35.3 ab | 57.7 a | 4.1 a | 1.7 ab | 1.2 a | 0.0 a |
| **L-0.4AUKI- II** | 33.5 b | 55.8 a | 3.0 a | 6.1 a | 1.4 a | 0.3 a |
| **L-0.4Hesperadin** | 35.8 ab | 56.2 a | 6.0 a | 0.0 b | 2.0 a | 0.0 a |

| | | | Tricellular structures | | Embryogenic structures | |
|---|---|---|---|---|---|---|
| **C) Pavon 10dC (%)** | **Microspores** | **Bicellular** | **Pollen-like** | **Embryogenic** | **Tetracellular** | **Multicellular** |
| **L-CM** | 57.2 a | 31.6 ab | 2.2 ab | 4.3 ab | 2.3 b | 2.3 d |
| **L-CM+DMSO** | 43.6 ab | 29.9 b | 0.0 b | 8.8 a | 7.8 ab | 9.8 bc |
| **L-0.4TSA** | 34.2 bc | 28.8 b | 3.8 ab | 11.3 a | 3.9 ab | 18.1 ab |
| **L-0.4Chaetocin** | 25.0 c | 38.0 ab | 5.0 a | 9.9 a | 8.7 ab | 13.5 ab |
| **L-0.4CARM1I** | 28.6 c | 32.0 ab | 6.1 a | 9.2 a | 10.3 a | 13.8 ab |
| **L-0.4AUKI- II** | 24.8 c | 34.0 ab | 0.0 b | 11.7 a | 7.8 ab | 21.8 a |
| **L-0.4Hesperadin** | 37.6 bc | 44.0 a | 7.0 a | 0.0 b | 6.9 ab | 4.6 cd |

**Supplementary Table S2:** Percentages of microspores, bicellular structures, tricellular structures (both pollen-like structures or embryogenic structures), tetracellular and multicellular embryogenic structures after application of 0.4 µM of TSA (L-0.4TSA), Chaetocin (L-0.4Chaetocin), CARM1 inhibitor (L-0.4CARM1I), Aurora Kinase Inhibitor II (L-0.4AUKI-II) and Hesperadin (L-0.4Hesperadin), during a 24-hour stress treatment in SM liquid medium. **A**) after 2 days of culture (2dC); **B**) after 4 days of culture (4dC); **C**) after 10 days of culture (10dC) in Caramba wheat cultivar. L-CM = control in SM liquid medium; L-CM+DMSO= control DMSO in SM medium with 1 % DMSO. Values followed by the same letter within each treatment are not significantly different ($p<0.05$) according to a Chi square test.

| | | | | **Embryogenic structures** | |
|---|---|---|---|---|---|
| **A) Caramba 2dC (%)** | **Microspores** | **Bicellular** | **Tricellular** | **Tetracellular** | **Multicellular** |
| **L-CM** | 27.0 a | 71.1 a | 1.7 a | 0.2 a | 0.0 a |
| **L-CM+DMSO** | 23.1 a | 74.2 a | 2.4 a | 0.4 a | 0.0 a |
| **L-0.4TSA** | 210. a | 75.4 a | 3.4 a | 0.3 a | 0.0 a |
| **L-0.4Chaetocin** | 31.2 a | 63.0 a | 5.1 a | 0.6 a | 0.0 a |
| **L-0.4CARM1I** | 25.0 a | 70.9 a | 4.1 a | 0.0 a | 0.0 a |
| **L-0.4AUKI- II** | 22.6 a | 74.0 a | 3.4 a | 0.0 a | 0.0 a |
| **L-0.4Hesperadin** | 32.7 a | 64.0 a | 3.1 a | 0.3 a | 0.0 a |

| | | | **Tricellular structures** | | **Embryogenic structures** | |
|---|---|---|---|---|---|---|
| **B) Caramba 4dC (%)** | **Microspores** | **Bicellular** | **Pollen-like** | **Embryogenic** | **Tetracellular** | **Multicellular** |
| **L-CM** | 41.1 a | 54.1 a | 4.5 c | 0.0 a | 0.3 a | 0.0 a |
| **L-CM+DMSO** | 39.8 a | 51.7 a | 6.7 bc | 0.8 a | 1.1 a | 0.0 a |
| **L-0.4TSA** | 23.1 b | 58.9 a | 16.1 a | 0.0 a | 1.2 a | 0.8 a |
| **L-0.4Chaetocin** | 36.5 ab | 56.1 a | 4.5 c | 2.3 a | 0.6 a | 0.0 a |
| **L-0.4CARM1I** | 30.1 ab | 55.9 a | 13.7 ab | 0.0 a | 0.4 a | 0.0 a |
| **L-0.4AUKI- II** | 32.4 ab | 61.3 a | 3.0 c | 3.0 a | 0.3 a | 0.0 a |
| **L-0.4Hesperadin** | 29.9 ab | 65.0 a | 5.1 c | 0.0 a | 0.0 a | 0.0 a |

| | | | **Tricellular structures** | | **Embryogenic structures** | |
|---|---|---|---|---|---|---|
| **C) Caramba 10dC (%)** | **Microspores** | **Bicellular** | **Pollen-like** | **Embryogenic** | **Tetracellular** | **Multicellular** |
| **L-CM** | 40.9 a | 40.0 ab | 0.0 c | 14.6 ab | 1.8 b | 2.7 ab |
| **L-CM+DMSO** | 41.5 a | 39.2 ab | 2.9 bc | 8.7 bc | 4.6 ab | 3.1 ab |
| **L-0.4TSA** | 32.0 ab | 49.2 a | 0.0 c | 9.4 bc | 4.7 ab | 4.7 ab |
| **L-0.4Chaetocin** | 40.7 a | 30.2 b | 0.0 c | 19.8 a | 8.1 a | 1.2 ab |
| **L-0.4CARM1I** | 40.8 a | 44.1 a | 9.9 b | 3.3 cd | 2.0 b | 0.0 b |
| **L-0.4AUKI- II** | 37.9 ab | 36.7 ab | 0.0 c | 11.2 ab | 8.7 a | 5.6 a |
| **L-0.4Hesperadin** | 24.6 b | 48.6 a | 20.3 a | 0.0 d | 4.4 ab | 2.2 ab |

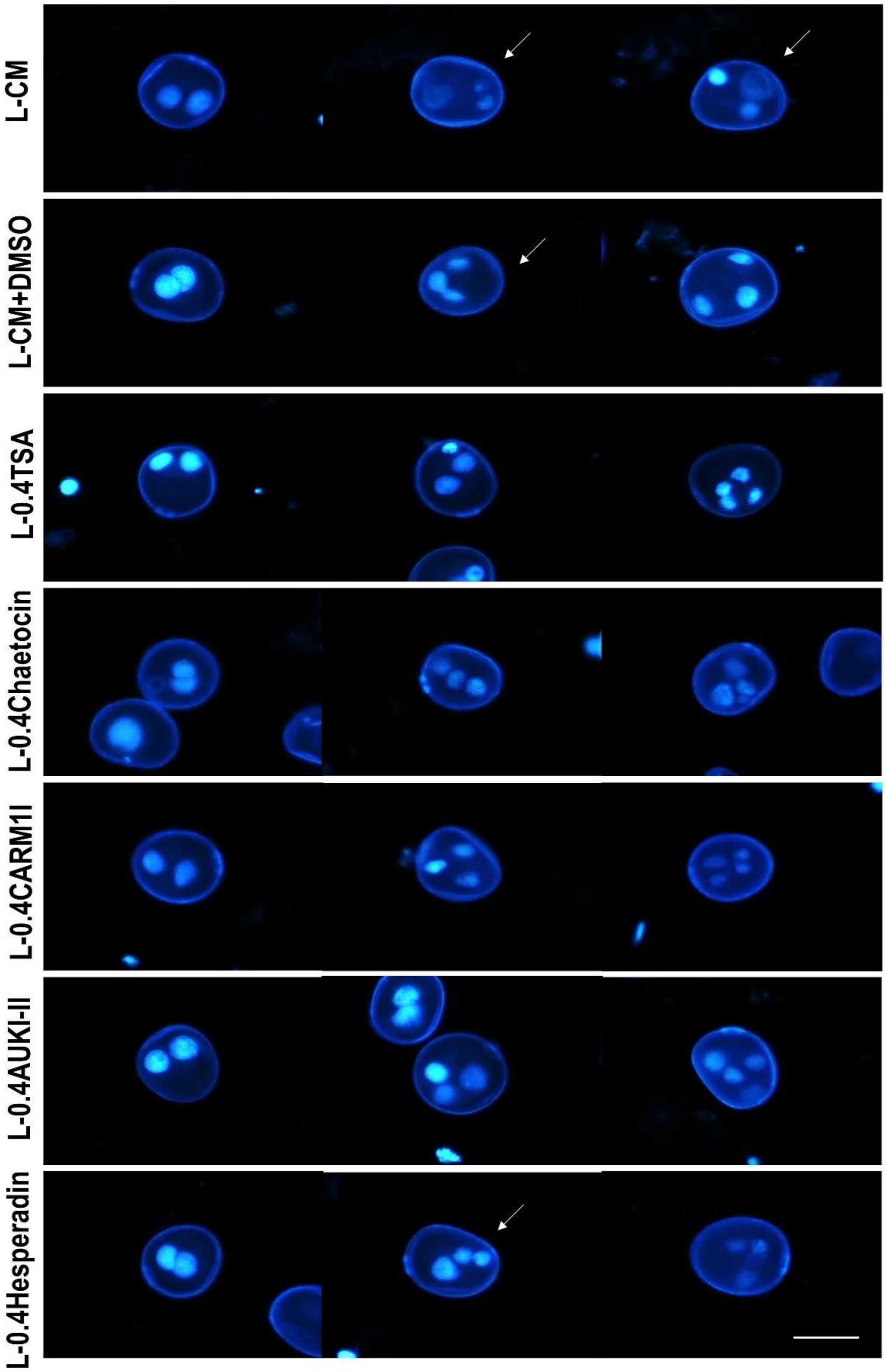


**Suplementary Figure S2**: DAPI staining of bicellular (both symmetric and asymmetric divisions), tricellular (both pollen-like and embryogenic), and tetracellular embryogenic structures after a 24-hour treatment in SM liquid medium with 0.4 μM TSA (L-0.4TSA), Chaetocin (L-0.4Chaetocin), a CARM1 inhibitor (L-0.4CARM1I), Aurora Kinase inhibitor II (L-0.4AUKI-II) and Hesperadin (L-0.4Hesperdin), in Pavon cultivar after 4 days in culture (4dC); L-CM = Control in SM liquid medium; L-CM+DMSO = Control DMSO in SM liquid medium with 1 % DMSO. White arrow: tricellular pollen-like structure. Scale bar = 20 μm.

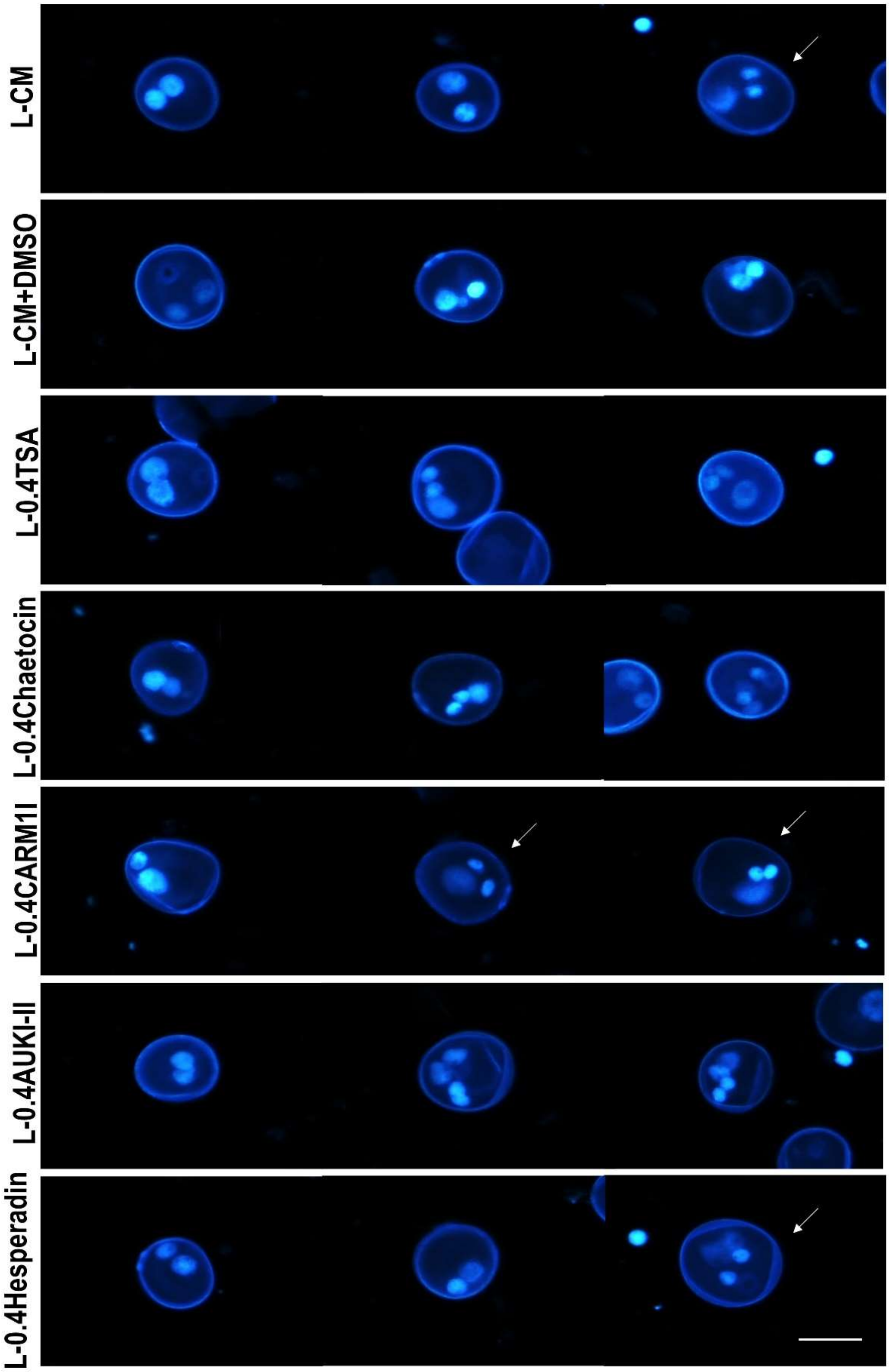


**Supplementary Figure S3**: DAPI staining of bicellular (both symmetric and asymmetric divisions), tricellular (both pollen-like and embryogenic) and tetracellular embryogenic structures after a 24-hour treatment in SM liquid medium with 0.4 μM TSA (L-0.4TSA), Chaetocin (L-0.4Chaetocin), a CARM1 inhibitor (L-0.4CARM1I), Aurora Kinase inhibitor II (L-0.4AUKI-II) and Hesperadin (L-0.4Hesperdin), in Caramba cultivar after 4 days in culture (4dC); L-CM = Control in SM liquid medium; L-CM+DMSO = Control DMSO in SM liquid medium with 1 % DMSO. White arrow: tricellular pollen-like structure. Scale bar = 20 μm.

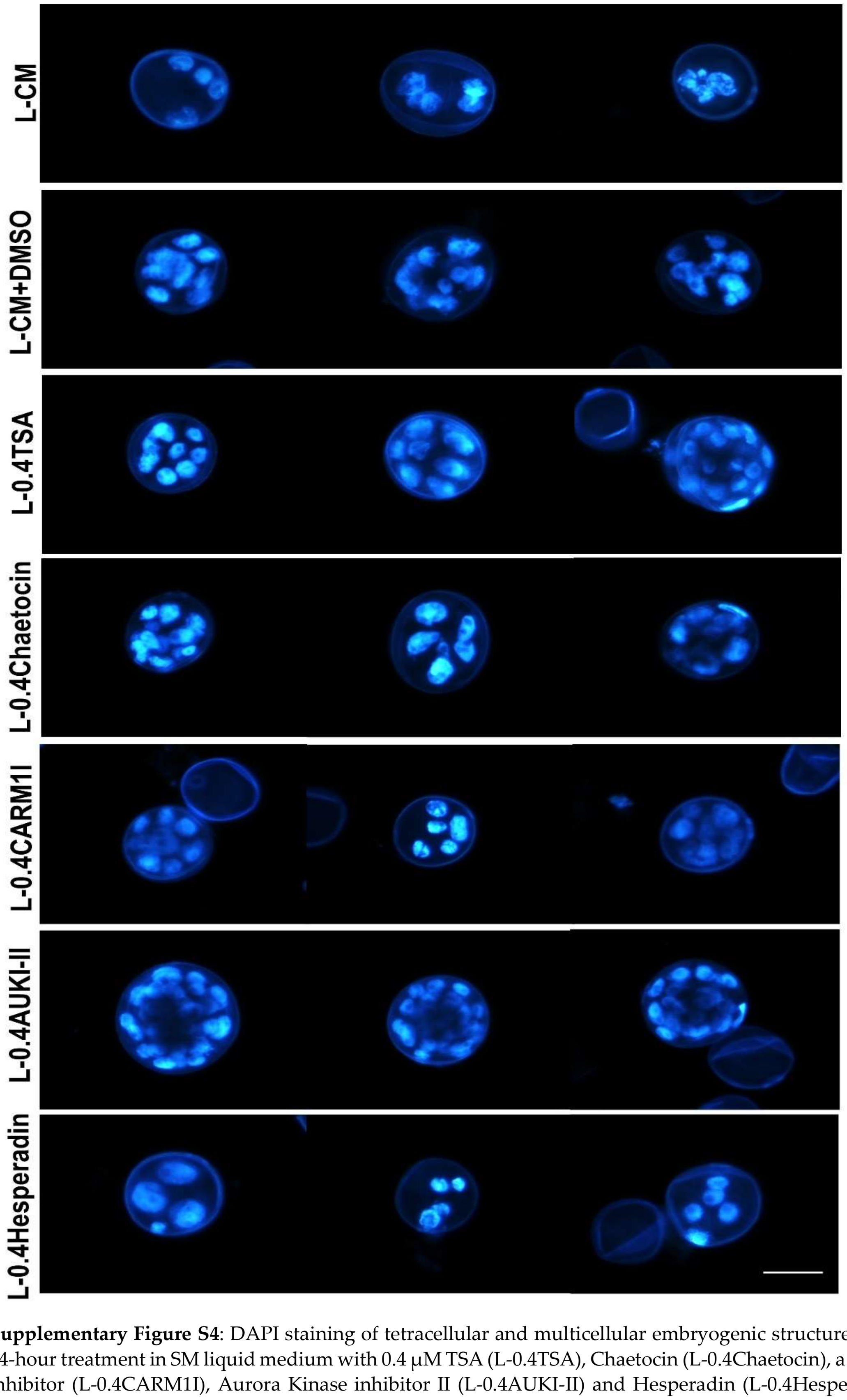


**Supplementary Figure S4**: DAPI staining of tetracellular and multicellular embryogenic structures after a 24-hour treatment in SM liquid medium with 0.4 μM TSA (L-0.4TSA), Chaetocin (L-0.4Chaetocin), a CARM1 inhibitor (L-0.4CARM1I), Aurora Kinase inhibitor II (L-0.4AUKI-II) and Hesperadin (L-0.4Hesperdin), in Pavon cultivar after 10 days in culture (10dC); L-CM = Control in SM liquid medium; L-CM+DMSO = Control DMSO in SM liquid medium with 1 % DMSO. Scale bar = 20 μm.

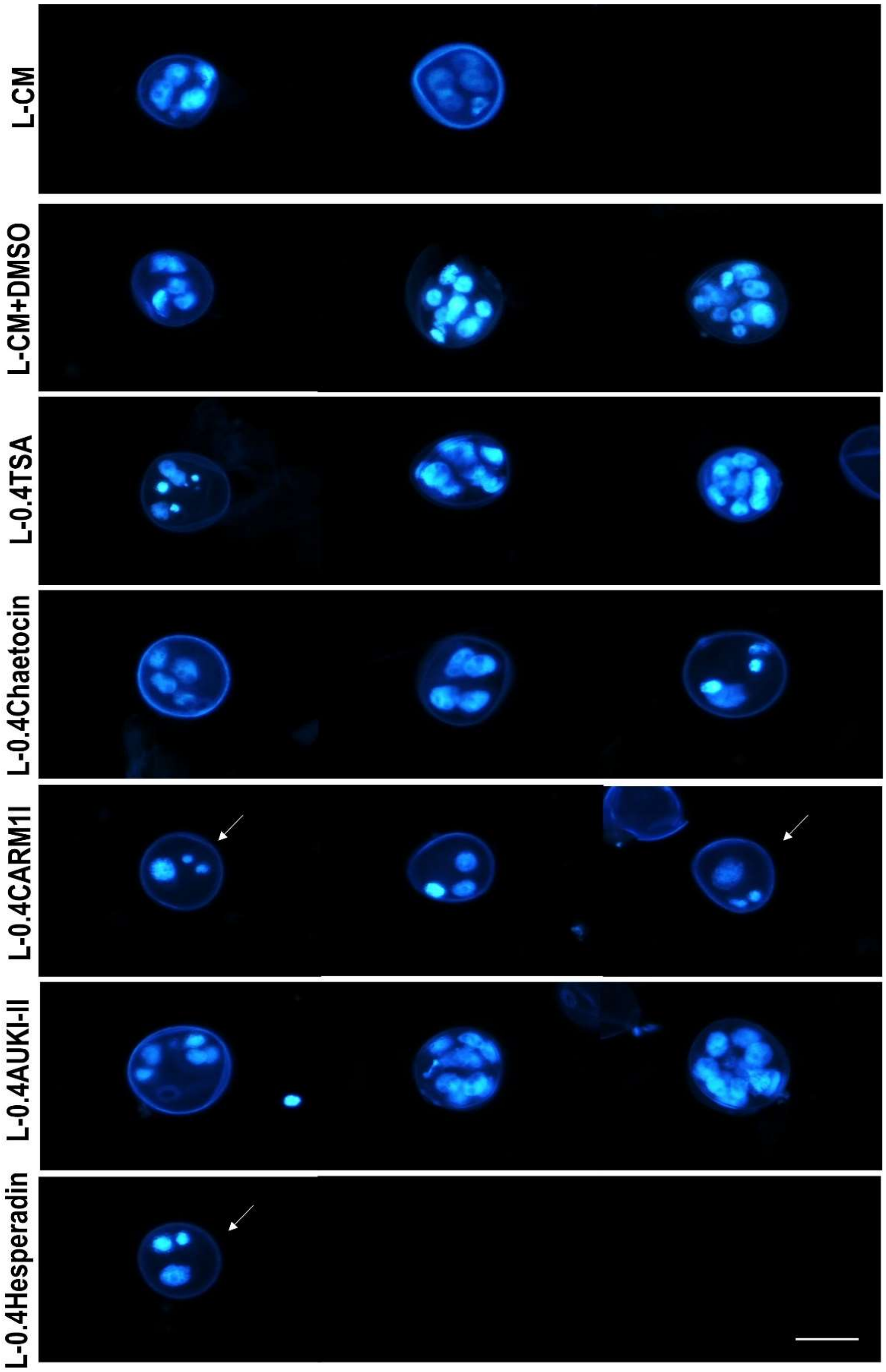


**Supplementary Figure S5**. DAPI staining of tricellular pollen-like, and tetracellular and multicellular embryogenic structures after a 24-hour treatment in SM liquid medium with 0.4 μM TSA (L-0.4TSA), Chaetocin (L-0.4Chaetocin), a CARM1 inhibitor (L-0.4CARM1I), Aurora Kinase inhibitor II (L-0.4AUKI-II) and Hesperadin (L-0.4Hesperdin), in Caramba cultivar after 10 days in culture (10dC); L-CM = Control in SM liquid medium; L-CM+DMSO = Control DMSO in SM liquid medium with 1 % DMSO. White arrow: tricellular pollen-like structure. Scale bar = 20 μm.
